# A182 THE LACK OF CHROMOGRANIN A IMPACTS COLONIC EPITHELIAL CELLS MARKERS IN AN EXPERIMENTAL MODEL OF ULCERATIVE COLITIS

**DOI:** 10.1093/jcag/gwab049.181

**Published:** 2022-02-21

**Authors:** D M Tshikudi, N Eissa, A Diarra, J Ghia

**Affiliations:** 1 University of Manitoba Faculty of Health Sciences, Winnipeg, MB, Canada; 2 Immunology, University of Manitoba, Winnipeg, MB, Canada; 3 Departement of Immunology, University of manitoba, Winnipeg, MB, Canada; 4 Immunology and Internal Medicine Section of Gastroenterology, University of Manitoba, Winnipeg, MB, Canada

## Abstract

**Background:**

Ulcerative colitis (UC) is a chronic and acute inflammatory disorder of the colon linked to dysregulated gut mucosal immune response and compromised colonic epithelial barrier function and integrity. Chromogranin-A (CHGA), a pro-peptide secreted by enteroendocrine cells, is highly expressed in colonic tissues of patients with UC. Elevated CHGA has been shown to correlate with UC disease activity and severity. Moreover, complete deletion of CHGA was shown to result in a diminution of pro-inflammatory markers known to disrupt the colonic epithelial barrier function and gut mucosal healing process. However, little is known about the effect of the absence of CHGA on colonic epithelial barrier function and gut mucosal integrity.

**Aims:**

Here, we characterized the impact of the lack of CHGA on the colonic mucosa and epithelial barrier structure and function using CHGA knockout (CHGA^-/-^) mice treated with dextran sulfate sodium (DSS) to induce colitis.

**Methods:**

13–17 weeks old male C57BL/6 wild-type (CHGA^+/+^) and C57BL/6 CHGA^-/-^ mice were treated for 5 days with 5% DSS to induced acute colitis, control mice received regular water. CHGA mRNA expression, disease activity index (DAI), and macroscopic score (MS) were analyzed. Distal colonic tissues were isolated, and mRNA expression of markers associated with regenerative stem cells (fast-cycling stem cells [Lgr5^+^] and reserve stem cells [HOPX^+^] and [LY6a^+^], Goblet cells functions mucus barrier mucin 2 [MUC2], resistin-like molecule β [RELMβ], WAP 4-disulphide core domain 2 [WFDC2]) and trefoil factor 3 (TFF3) was evaluated by qRT-PCR.

**Results:**

We validated a beneficial effect of the Lack of CHGA on colitis severity, associated with significantly lower DAI and MS. In colitic CHGA^+/+^ and CHGA^-/-^ mice, Lgr5^+^ and HOPX^+^ were both highly down-regulated, although, compared to CHGA^+/+^, CHGA^-/-^ mice presented a 10.5fold higher expression of HOPX^+^. Compared to non-colitic states, Ly6a^+^ expression was significantly elevated in both colitic CHGA^+/+^ and CHGA^-/-^ mice, however, no differences in Lgr5^+^ and Ly6a^+^ expression were noted between CHGA^+/+^ and CHGA^-/-^ mice in all conditions. In CHGA^+/+^ mice, inflammatory conditions led to higher MUC2 and RELMB expression, although, compared to CHGA^+/+^, these markers were significantly lower in CHGA^-/-^ mice. In colitic conditions, compared to CHGA^+/+^, CHGA^-/-^ had a significant increase of WFDC2. In non-colitic conditions, mRNA expressions of all markers evaluated between CHGA^+/+^ and CHGA^-/-^ in this study were unaltered. Finally, no differences were observed in TFF3 gene expression.

**Conclusions:**

These results indicate in the absence of CHGA, the colonic epithelial barrier integrity and function are maintained through the modulation of goblet cells functions and elevated gut mucosa regenerative potential, thus enhancing the mucosal protection to colitis damage.

**Funding Agencies:**

CIHR

